# Anticancer Properties of Eugenol: A Review

**DOI:** 10.3390/molecules26237407

**Published:** 2021-12-06

**Authors:** Ali T. Zari, Talal A. Zari, Khalid Rehman Hakeem

**Affiliations:** Department of Biological Sciences, Faculty of Science, King Abdulaziz University, P.O. Box 80203, Jeddah 21589, Saudi Arabia; azari@kau.edu.sa (A.T.Z.); talzari@yahoo.com (T.A.Z.)

**Keywords:** eugenol, clove oil, antioxidant, anti-inflammatory, anticancer properties, apoptosis, proliferation, migration, invasion, metastasis, autophagy

## Abstract

Conventional cancer treatments have shown several unfavourable adverse effects, as well as an increase in anticancer drug resistance, which worsens the impending cancer therapy. Thus, the emphasis is currently en route for natural products. There is currently great interest in the natural bioactive components from medicinal plants possessing anticancer characteristics. For example, clove (*Syzygium aromaticum* L.) (Family Myrtaceae) is a highly prized spice that has been historically utilized as a food preservative and for diverse medical uses. It is reckoned amongst the valued sources of phenolics. It is indigenous to Indonesia but currently is cultivated in various places of the world. Among diverse active components, eugenol, the principal active component of *S. aromaticum*, has optimistic properties comprising antioxidant, anti-inflammatory, and anticancer actions. Eugenol (4-allyl-2-methoxyphenol) is a musky oil that is mainly obtained from clove. It has long been utilized all over the world as a result of its broad properties like antioxidant, anticancer, anti-inflammatory, and antimicrobial activities. Eugenol continues to pique investigators’ interest because of its multidirectional activities, which suggests it could be used in medications to treat different ailments. Anticancer effects of eugenol are accomplished by various mechanisms like inducing cell death, cell cycle arrest, inhibition of migration, metastasis, and angiogenesis on several cancer cell lines. Besides, eugenol might be utilized as an adjunct remedy for patients who are treated with conventional chemotherapy. This combination leads to a boosted effectiveness with decreased toxicity. The present review focuses on the anticancer properties of eugenol to treat several cancer types and their possible mechanisms.

## 1. Introduction

Globally, cancer is recognized as the second principal reason of demise. In 2018, it was accountable for about 9.6 million deaths. Around one in six deaths internationally is as a result of cancer [1]. Cancer is a general term for many ailments that can influence any part of the body. Cancer is a prominent reason for deaths globally and was responsible for killing approximately 10 million people in 2020 [2]. One of the record mutual traits of cancer is the quick formation of aberrant cells that expand beyond their normal borders, allowing them to infect neighboring sections of the body and migrate to other tissues; this course is known as metastasis. It is the chief reason for death due to cancer [3].

Medicinal plants have been utilized for a long time as sources of traditional remedies in addition to acting as a basis of modern medicine. Because of the efficiency, low cost, and few side effects linked with medications developed from medicinal plants, they are generating a lot of interest. Various components with pharmacological characteristics have been found in medicinal plants [4,5,6]. Plants produce essential oils as secondary metabolites, which are complex combinations of volatile compounds. These essentials oils are extremely volatile, liquid, and dissolve in lipids and a range of organic solvents [7,8]. Bioactive terpenes (like mono/sesquiterpenes) and aromatic molecules are present in varied proportions in essential oils. They possess antioxidant [9,10], anti-inflammatory, and antimicrobial properties [11,12], which make them very interesting for chemoprevention purposes since chronic inflammation is frequently associated with carcinogenesis [13,14,15,16].

Clove (*Syzygium aromaticum* (L.) (Family Myrtaceae) is considered a very important herb in traditional medicine, having extensive biological activities. Phytocomponents of clove consist of many classes and groups of chemical compounds like hydrocarbon, monoterpenes, phenolics, and sesquiterpenes compounds. Eugenol (70–85%), eugenol acetate (15%), and β-caryophyllene (5–12%) are the most common phytochemicals identified in clove oil [17]. Among different active ingredients, eugenol (4-allyl-2-methoxyphenol) has many biological benefits like antioxidant, anticarcinogenic, antibacterial, antifungal, and insecticidal activities. Apart from its widespread use as a food flavoring agent, clove oil has long been utilized as a topical analgesic in dentistry [17]. Eugenol is a phenolic aromatic ingredient that is chiefly derived from clove oil. It was originally identified as an aromatic component from *Eugenia caryophyllata* in 1929 and then provided commercially in the United States in 1940 [18,19]. Eugenol has a lot of applications owing to its many characteristics. Furthermore, it is a possible component of diverse therapeutic products, including those planned to cure human cancers, as a result of the increasing interest in traditional medicines that comprise natural components [20,21]. Furthermore, isoeugenol derivatives have come to be a general research topic owing to their valuable benefits, such as antimicrobial, pesticidal, and antitumor activities [19].

Therefore, eugenol has been generally investigated for its varied properties like anti-inflammatory and antioxidant effects [22,23]. Moreover, it is presently recognized for its actions against several human cancer types, whereas it has insignificant toxicity towards normal cells. Several studies have displayed diverse molecular pathways for varied curative mechanisms of eugenol in the treatment of various cancer types [23,24,25,26,27]. Anticancer effects of eugenol are accomplished by various mechanisms like apoptosis induction, cell cycle arrest, inhibition of proliferation, migration, angiogenesis, and metastasis on several cancer cell lines [28,29,30,31]. Besides, eugenol might be utilized as an adjunct therapy for patients who are treated with conventional chemotherapy. This combination leads to a boosted effectiveness with decreased toxicity [32,33]. Therefore, the current review emphasizes the anticancer properties of eugenol to treat several cancer types.

## 2. Materials and Methods

Databases like Science Direct, PubMed, Google Scholar, and Scopus were explored for the terms: clove, *Syzygium aromaticum*, *S. aromaticum*, eugenol and their anticancer, antioxidant, anti-inflammatory activities, eugenol apoptosis, proliferation, migration, invasion, metastasis, angiogenesis, autophagy, eugenol cancer combinations, and eugenol cancer types between the years 2010 and September 2021 to prepare the current review.

## 3. Eugenol Characteristics

**Figure 1:**
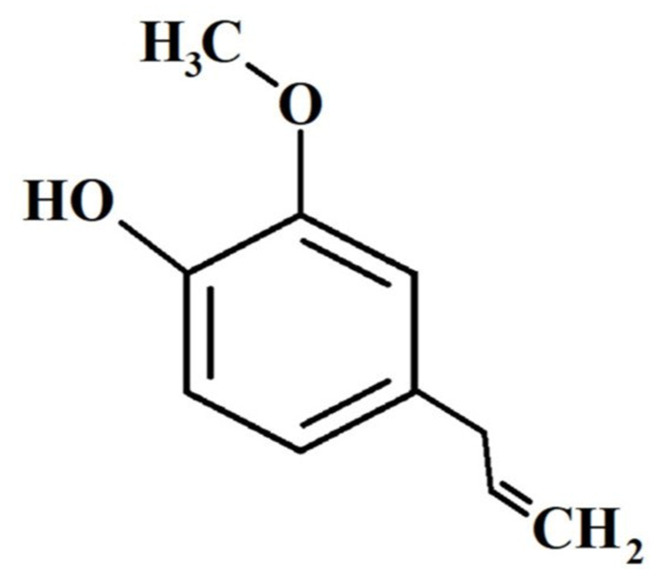
Eugenol’s chemical structure.

Eugenol (4-allyl-2-methoxyphenol; C_10_H_12_O_2_) is considered an aromatic ingredient, which belongs to the phenol group (Figure 1). Eugenol is the foremost constituent in clove oil (*Syzygium aromaticum*) and is generally derived from plant essential oils of several families, such as Myrtaceae, Lauraceae, Lamiaceae, and Myristicaceae. Though it is recognized to happen in varying amounts depending on the species, *S. aromaticum* is the richest source and its concentrations range from 9.38 to 14.65 g/100 g fresh plant, and is mainly accountable for its distinguishing aroma [19,34,35,36,37].

The concentration of eugenol present in clove oil varies from 70% to 96% [38]. It comprises various functional groups, like allyl (-CH_2_-CH=CH_2_), methoxy (−OCH_3_), and phenol (OH) [39].

Eugenol is an aromatic pale yellowish liquid that dissolves well in organic solvents and moderately in water. Two methods are synthetically used to form eugenol: one method includes guaiacol allylation with allyl chloride. The technique depends on the biotransformation of many microorganisms, like *Streptomyces* spp., *Escherichia coli*, and *Corynebacterium* spp. [19,35,36,37].

The chemical stability of eugenol is low. Eugenol is susceptible to oxidation and many biochemical interactions. It is quickly absorbed via diverse organs and processed in the liver when taken orally. Thus, encapsulation of eugenol appears to be the finest approach for avoiding early absorption, improving its water solubility, and, therefore, increasing its action. For example, it has been stated that when eugenol is supplied as solid lipid nanoparticles, the quantity of eugenol delivered to infected cells upsurges by at least sixfold [19]. The eugenol inclusion complexes might boost the heat stability, allowing for slower eugenol release. This could be eugenol microemulsions made by dissolving its essential oil (0.75–1.5 percent *w*/*w*) in surfactant micelles [19,40].

Eugenol displays diverse antioxidant, anti-inflammatory, antimutagenic, antiallergic, analgesic, and antitumor characteristics. Besides, it has exhibited antimicrobial influences against various human diseases, such as varied fungi, bacteria, and several parasites, like *Fasciola gigantica*, *Giardia lamblia*, and *Haemonchus contortus*. Moreover, eugenol can take care of the liver from the hepatotoxic effects of carbon tetrachloride (CCl_4_) [19,21,34,36,41].

Eugenol is well-known for its antibacterial properties. For this reason, it has been extensively used for oral and dental care. It reduces local pain and has disinfecting activities. In dental medicine, amorphous chelate compounds made from eugenol in conjunction with zinc oxide are used for covering the pulp indirectly. It is also utilized to fill root canals in liquid form in particular pastes. Occasionally, eugenol is put on the gums for numbing prior to dentures being introduced [18,34,42,43].

**Figure 2:**
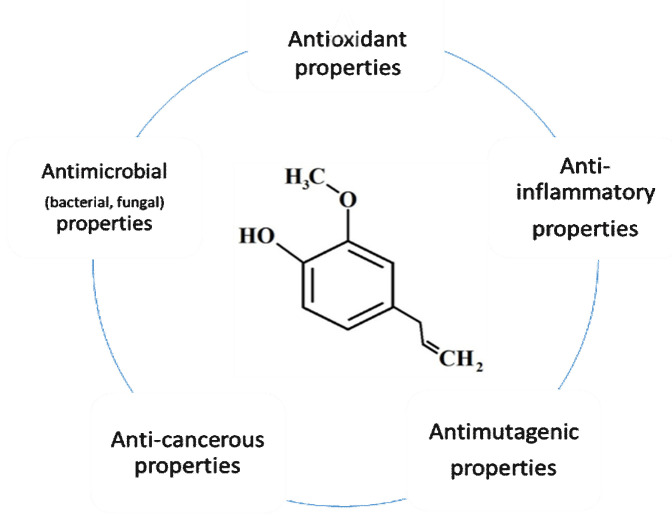
General applications of eugenol.

Eugenol is usually utilized in medications, cosmetics, food, and as a local analgesic and antiseptic at low concentrations and has numerous actions (Figure 2). Furthermore, eugenol is a general component that is found in a variety of goods around the house, like soaps, fragrances, and skincare products. Additionally, it is utilized as a pesticide, fumigant, and preservative to keep foods safe from microbes. According to the Joint FAO/WHO Expert Committee on Food Additives (JECFA), the maximum daily dose of eugenol for humans is 2.5 mg/kg body weight [18,21,37,42].

Eugenol is utilized to treat gastrointestinal and respiratory contagions. Furthermore, it comprises many medicines that have been suggested for the treatment of upper respiratory mucosa inflammation and cold prevention. These medicines are generally administered as inhalation and aerosol remedies, such as Aromatol, Amol, or Olbas [43].

Eugenol has applications in food manufacturing and agriculture as a result of its diverse activities, such as antimicrobial. Its valuable influence is linked with low doses of efficient action, which is a significant benefit. Furthermore, eugenol is efficient against numerous foodborne pathogens [19,40]. Eugenol is also utilized as a biocontrol in agriculture due to its potential to decrease *Salmonella* infection of organic products via inhibition of its growth in soil. Furthermore, its antifungal characteristics are used to preserve fruits like apples, strawberries, and peaches, as well as their juices, from hazardous microbes [40].

Although excessive amounts of eugenol can cause harmful effects, a concentration of 2.5 mg/kg body weight is commonly considered harmless. Due to its use in dentistry, there are cases of hand eczema, in addition to burning mouth syndrome (BMS) or allergic contact gingivitis [44].

## 4. Eugenol as an Antioxidant

Eugenol is considered a general antioxidant. It does inhibit monoamine oxidase. It has been shown to have neuroprotective characteristics [45]. It is acknowledged to guard microbial DNA and proteins from damage, stop the generation of reactive forms of nitrogen, sift free radicals, increase the cyto-antioxidant capacity, and hinder the manufacture of reactive oxygen species. Furthermore, eugenol can remove damaged molecules, stop cancer-causing mutations, and repair oxidative damage [18,36,42]. The antioxidant capabilities of eugenol are attributed to its configuration, as its structure permits the repairing of free radicals by accepting supplied hydrogen atoms [46].

The capability of specific constituents to give hydrogen, particularly those containing a phenolic group in their structure, accounts for the influence of the free radical scavenger of 2,2-diphenyl-1-picrylhydrazyl (DPPH) [47]. Both eugenol and clove oil display potent antioxidant capabilities. They possess a potent DPPH radical scavenging influence (half maximal inhibitory concentration (IC_50_) = 11.7 μg/mL for eugenol; 13.2 μg/mL for clove oil) and hinder reactive oxygen species (ROS) generation in human neutrophils activated via phorbol 12-myristate 13-acetate (IC_50_ = 1.6 μg/mL for eugenol; 7.5 μg/mL for clove oil) or H2O2 (IC_50_ = 27.1 μg/mL for eugenol; 22.6 μg/mL for clove oil). In addition, they hinder the generation of nitric oxide (IC_50_ = 19.0 μg/mL for eugenol; 39.8 μg/mL for clove oil) and reveal elevated myeloperoxidase (MPO) hindrance in human leucocytes (IC_50_ = 19.2 μg/mL for eugenol; 16.3 μg/mL for clove oil) [48]. When the concentration of eugenol was lowered from 1.0 to 0.1 μM/mL, eugenol was capable of removing approximately 81% of the DPPH radicals and decreasing the power of radicals [49].

Clove and eugenol displayed similar antioxidant actions, with standards of seizing radicals DPPH and ABTS, respectively, IC_50_ = 0.3257 and 0.1595 mg/mL for clove, and IC_50_ = 0.1967 and 0.1492 mg/mL for eugenol. Thus, the antioxidizing character of clove essential oil is linked to its eugenol content [50]. A study that linked eugenol’s antioxidant and anti-inflammatory accomplishments was conducted to validate the biochemical profile of this component. Yogalakshmi et al. demonstrated that exposure of rats to eugenol (10.7 mg/kg body weight/day) for 15 days reduced the translation of inflammatory markers (IL-6, COX-2, and TNF-α), lipid peroxidation indices, and protein oxidation [51]. Kaur et al. confirmed these findings in male mice. This indicates that eugenol acts as both an anti-inflammatory and an antioxidant agent [52]. Pretreatment with eugenol was capable of dramatically enhancing SOD1, CAT, Gpx1, and GST levels as well as decreasing inflammation triggered via lung exposure to LPS. Therefore, eugenol can protect against the damage produced via oxidative stress and can also be employed as an anti-inflammatory drug [53].

Although eugenol is recognized to possess antioxidant and anti-inflammatory activities at small dosages, at greater concentrations, it can have a pro-oxidative influence, causing the production of free radicals. Furthermore, DNA disintegrated in normal human fibroblast cells can be raised under the influence of clove oil at high concentrations, according to several studies [18,37,54,55].

## 5. Eugenol Anti-Inflammatory Agent

Inflammation is defined as a complicated protective reaction of the body against hazardous causes like microbes or damaged cells [56,57], with the biological system’s goal being to eliminate toxic stimuluses from the body along with enhancing the cure. Nevertheless, this reaction must be regulated, and only endure for a brief length of time; otherwise, it may lead to the development of immune-related pathologies [58]. Inflammation is usually classified as either acute or chronic. The former is a type of innate resistant response distinguished by occupant cell stimulation, the release of proinflammatory cytokines and chemokines, and the influx of polymorphonuclear cells, usually polymorphonuclear neutrophils, to damaged places. This response complex boosts the basic indicators of inflammation, including heat, pain, and edema [59]. However, the latter one is a protracted response distinguished via a steady alteration in cell types found at the inflammatory place, which results in repair and overcomes the damage. Elevated blood streaming through dilation of blood vessels and the release of proinflammatory intermediaries occur in both forms of inflammation [57,60].

The nuclear factor-kappa B (NF-κB) signaling pathway performs a substantial part in the immune response. It has significance in inflammatory procedures owing to its role in cytokine transcription, like nitric oxide (NO) and others. The pharmaceutical industry is interested in substances that block this pathway, such as eugenol [33,61,62]. Commonly, patients with inflammatory ailments utilize glucocorticoids or nonsteroidal anti-inflammatory drugs (NSAIDs). Nevertheless, such medicines are linked to serious side effects, such as bleeding and digestive ulcers. Additionally, restricted remedial effectiveness was observed by these medicines, which frequently causes patients to discontinue treatment [63]. Therefore, the pharmacological industry has focused efforts to discover novel bioactive compounds.

The bioactive ingredients from medicinal plants are appealing for the progress of novel medicines that target various ailments, like those linked to inflammatory actions, that are often connected to oxidative stress. The majority of these ingredients can inhibit oxidative stress and inflammatory responses. In addition, these compounds might participate in a protective method for improving life quality through the intake of a diet rich in them [22].

The indication implies that eugenol possesses the capability to hinder the making of superoxide anions in neutrophils by obstructing the Raf/MEK/ERK1/2/p47-phosphorylation pathway. Furthermore, it is recognized as a suppressor of proinflammatory intermediaries, comprising tumor necrosis factor-alpha (TNF-α), IL-1β and IL-6, prostaglandin E2 (PGE2), inducible oxide nitrate synthase (iNOS) expression, and expression of cyclooxygenase-2 (COX-2), leukotriene C4 and 5-lipoxygenase (5-LOX), and nuclear factor kappa B (NF-κB) [64]. The anti-inflammatory action of eugenol is linked with stopping neutrophil/macrophage chemotaxis and hindering the production of inflammatory neurotransmitters, like leukotrienes and prostaglandins. Moreover, its dimers have demonstrated chemoprotective activities via hindering macrophage cytokine expression [21,45].

Eugenol has extensive pharmacological properties and action on the redox condition. It is also utilized in the food and pharmacological commerce. In terms of eugenol’s importance, Barboza et al. reviewed its antioxidant and anti-inflammatory physiognomies, besides its methods of action and remedial capability to treat inflammatory ailments in vitro/in vivo [22]. Eugenol prevented the 7,12-dimethylbenz[a]anthracene (DMBA) and 12-O-tetradecanoylphorbol-13-acetate (TPA) stimulated skin carcinogenesis. Eugenol’s anti-inflammatory characteristics are linked to its molecular mechanism, because of decreased levels of proinflammatory cytokines like IL-6 and TNF-, as well as inflammation enzyme markers like COX and iNOS, which are linked to redox status modulation with decreased MDA and elevated antioxidative enzymes. Therefore, these findings potently imply the chemotherapeutical capacity of eugenol against carcinogenesis [22].

Eugenol has been linked to a number of pharmacologic actions, such as antipyretic [65], anticancer [66], anesthetic [67], anti-inflammatory [68], analgesic [69], and antimicrobial [70,71,72]. Eugenol is a common pain reliever and anesthetic utilized in dentistry. Varied studies have observed that it hinders voltage-regulated Na^+^ channels (VRSCs) in the mainstream neurons of the teeth [73,74].

Continuing research has demonstrated that eugenol hinders COX-2 and 5-LOX [67]. As a result, eugenol could potentially work as an anti-inflammatory compound, permitting it to substitute certain NSAIDs in varied ailments. Furthermore, it might be utilized in the development of novel eclectic medicines to fight disorders linked with inflammatory procedures, like cancer or osteoarthritis.

## 6. Eugenol Anticancer Properties

Eugenol has exhibited anticancer properties against several cancer types, such as leukemia, lung cancer, colon cancer, colorectal cancer, skin cancer, gastric cancer, breast cancer, cervical cancer, and prostate cancer [23,24,25,26,27] (Table 1). Yoo et al. assessed eugenol’s efficacy against human promyelocytic leukemia cells (HL-60). Eugenol triggered cell apoptosis in these cancerous cells through a process reliant on elevated ROS production and decreased the mitochondrial membrane potential, indicating that it might possess apoptosis-triggering characteristics [75].

### 6.1. Lung Cancer

Additionally, indication implies that eugenol may act as both an antioxidant, stopping mutation, and a pro-oxidant in cancer cells, affecting signal pathways and destroying tumor cells. The molecular process is thought to comprise varied phases: decreasing cyclooxygenase-2 activity, hindering NF-κB stimulation, downregulation of prostaglandin production, activating arrest of cell cycle S-phase, and triggering apoptosis by reducing inflammatory cytokine levels [20,55]. Chemotherapeutic activities were observed in eugenol against human lung cancer based on the Fangjun and Zahijia study [20]. Through inhibition of the PI3K/Akt pathway and prevention of MMP (matrix metalloproteinase) action, an in-laboratory study using lung cancer adenocarcinoma cells A549 and human embryonic lung fibroblast MRC-5 demonstrated that a low concentration of this compound inhibited carcinogenic cell migration and incursion, hampered lung cancer cell sustainability, and stopped metastasis. At greater concentrations (1000 μM), eugenol exhibited lethal influences on normal and lung cancer cells. Lung cancer has become one of the principal reasons for death worldwide in males. Furthermore, it is increasing in females at a disturbing degree. The goal of the research of Choudhury et al. was to utilize eugenol properties to restrict cancer progress in NDEA-triggered lung carcinogenesis. Eugenol was used to find the chemo-preventive mechanism behind the NDEA-triggered mouse lung carcinogenesis model in a vital efficient method and was confirmed in the A549 human lung cancer cell line. Choudhury et al. [30] explored the drug-resistant and the strongest cancer cells called CSCs by investigating their controller molecule β-catenin. The nontoxic concentration of eugenol was demonstrated to induce apoptosis, concurrently repressing cell invasion in the lung tissue of carcinogen-treated mice while it did not influence the normal animals. Coalescing cellular apoptosis and proliferation, eugenol exhibited an outstanding chemoprotective capability in this lung cancer. It powerfully constrained the lung carcinoma in the trifling dysplastic stage as a chemoprotective agent. The molecular investigation greatly showed β-catenin nuclear transportation restriction. The diminished β-catenin pool and activated N-terminal Ser37 phosphorylation form after eugenol administration caused its cytoplasmic degradation. Subsequently, CSC markers like Oct4, CD44, EpCAM, and Notcht1, whose expression is reliant on β-catenin, were considerably reduced, as confirmed through ICC, IHC, and WB analysis both in vivo and in vitro. The in vitro secondary sphere-forming assay also verified the significantly suppressed CSC population, and thus the virulency. Furthermore, eugenol was verified to greatly boost β-catenin degradation after exposure to the CK1α inhibitor D4476 in vitro via Western blot. CK1α in the Wnt/β-catenin pathway has a vital function for labelling with the N-terminal Ser45 phosphorylation of β-catenin, which eventually unlocks a spot for critical phosphorylation via the means of GSK3β at the Ser37 residue to happen. Therefore, the decisive killing of CSCs was linked with reappearance owing to the handling failure. This may accomplish an extended and healthier life quality using a natural cheap method [30].

### 6.2. Colon Cancer

Essential oils are a complicated blend of volatile and hydrophobic components produced from aromatic plants, usually found in our food. Various studies have presently implied probable anticancer effects of several essential oil compounds. Essential oils show their anticancer potential against many human neoplastic cell lines, either alone or in combination with anticancer medicines [58]. Petrocelli et al. used cinnamaldehyde (essential oil obtained from cinnamon) and eugenol (essential oil obtained from bud clove) in his study on NCM-460 cells (epithelial colon) and anticancer action against colorectal cancer (CRC) cell lines [25]. Petrocelli et al. [25] distinguished cinnamaldehyde and eugenol as ingredients with a definite antitumor activity selectively targeting the transformed colonic cells. After 72 h of treatment, cell death, necrosis, and cell cycle slowing were activated by both cinnamaldehyde (75 µM) and eugenol (800 µM) in Caco-2 and SW-620 cells but not by NCM-460 cells. These two ingredients can possibly prevent or cure colorectal cancer if they are linked with targeted delivery to the colon.

This compound powerfully decreased the vitality of CRC cells and mere cytotoxic influences on healthy cells. The influence of eugenol (800 µM) was inspected in cells after 72 h. Eugenol inhibited G2 in Caco-2 cells, while it favored G1 accumulation in SW-620 cells. Eugenol showed no effect on the cell cycle of NCM-460. Such findings displayed that a specific dosage of eugenol can decline the proliferation of CRC cells by either shrinking the G2 phase or promoting the G1 phase. Besides, the Annexin V-FITC assessment via flow cytometry exhibited a substantial rise in necrosis and late apoptosis in CRC cells exposed to eugenol and did not affect NCM-460. The absence of influences in normal cells implies that eugenol can be used for CRC remedies or to prevent cancer. Furthermore, eugenol was effectual as an anticancerous agent under lab situations, given alone, or combined with conventional medicines such as doxorubicin [76,77]. The superior role of cinnamaldehyde against SW-620, regarding Caco-2 cells, shows its tremendous potential for metastases. Regrettably, this potential fades upon increasing the duration of treatment [25]. One more remarkable characteristic of cinnamaldehyde and eugenol is their display of antioxidant and pro-oxidant action in varied circumstances. Antioxidant potential has been designated as a defensive counter to carcinogenesis. After the establishment of cancer, the pro-oxidant influences can activate death of cancer cells through cell-signaling [39,89]. Moreover, the comparatively longer time duration required for the transition of a normal colon epithelial cell to become cancerous motivates us to test eugenol and cinnamaldehyde in an appropriate preparation to interrupt and decrease CRC instigation. However, the formulation of chemotherapeutical drugs and cinnamaldehyde and eugenol, previously exhibited, may also provide a novel pharmacologic chance to expand the anticancer influences in stubborn CRC types, redeeming the healthy colon epithelial cells.

### 6.3. Gastric Cancer

The combination of apoptosis stimulation and inhibition of cancer cell proliferation and angiogenesis conduct may be useful for cancer chemoprevention. Manikandan et al. [78] assessed the chemopreventive influences of eugenol on N-methyl-N′-nitro-N-nitrosoguanidine (MNNG)-triggered gastric cancer in Wistar rats. MNNG-treated rats established gastric carcinogenesis, showing avoidance of apoptosis along with promotion of proinvasive and angiogenic elements. Eugenol administration triggered apoptosis by the mitochondrial conduit through modulation of Bcl-2 family proteins, Apaf-1, cytochrome C, and caspase, and hindering cell proliferation and angiogenesis, as proved via alternations in MMPs actions and MMP-2 and -9, VEGF, VEGFR1, TIMP-2, and RECK expression. Therefore, plant secondary metabolites like eugenol, which can manipulate the balance of pro and antiapoptotic proteins, besides the subtle equilibrium of initiators and inhibitors of angiogenesis and invasion, are desirable contenders for stopping cancer development [78].

Modulation of the intracellular signaling pathway of NF-κB included in the downregulation of cell invasion and cell cycle monitoring molecules is a practical method of chemoprevention. Eugenol is recognized to have interesting healing properties. Manikandan et al. examined the modulating influences of eugenol on NF-κB signaling in gastric carcinoma due to N-methyl-N′-nitro-N-nitrosoguanidine (MNNG) in a rat model by analyzing the expression of NF-κB family members ((NF-κB, p50, and p65), kappaB alpha inhibitor (IκBα), phosphorylated IκBα (p-IκBα), IκB kinase β (IKKβ)), and the NF-κB target genes that enhance (e.g., cyclin D1, cyclin B, and PCNA) or hinder (e.g., p21, p53, and Gadd45) cell proliferation and cell survival. MNNG-triggered gastric tumors were distinguished by activation of NF-κB, which was associated with the upregulation of IKKβ and the phosphorylation and disintegration of IκBα. Moreover, the upregulation of cyclins and PCNA with the downregulation of p21, p53, and Gadd45 implied that the proliferative benefit in gastric carcinomas depends on the activity of NF-κB. Eugenol treatment greatly decreased the occurrence of MNNG-triggered gastric tumors by overpowering NF-κB stimulation and modulation of the translation of NF-κB target genes that control cell proliferation and cell survival. The modification of the NF-κB signaling pathway via eugenol can have a substantial effect on chemopreventive and remedial methods of cancer [79].

### 6.4. Cervical Cancer

Cervical cancer is a general gynecological tumor that mostly leads to death in women [90]. It results from sustained HPV type 16 and 18 infections. It is a metastatic tumor owing to its ability to attack and grow at distant places and result in metastases. Therefore, the chief cause of the high prevalence and mortality of cervical cancer is metastasis to other body parts. The epithelial-mesenchymal transition (EMT), particularly EMT type 3, is involved in cervical cell carcinoma metastasis [91,92]. Snail-1 (zinc-finger transcription factor that is controlled via the PI3K/Akt signaling pathway) increases EMT through downregulation of E-cadherin and upregulation of vimentin [93,94]. EMT causes epithelial cells to transform into mesenchymal cells, triggering damage of cell adhesion and polarity [95]. Migration ability, invasion, apoptosis resistance, and extracellular matrix formation all increase as a result of these changes in features [96].

Recently, chemotherapy is the most used treatment for metastatic cervical cancer; however, it can have the most serious side effects since it proves toxic to other normal cells surrounding the tumor, leading to necrosis. Furthermore, the fee of chemotherapy treatment is excessive [97]. Thus, treatments that are both more effective and less expensive are required. Permatasari et al. showed that eugenol’s antitumor mechanisms trigger cell death in cancer cells via activation of p53 protein levels [98]. To evaluate the antimetastatic properties of eugenol against cervical cancer, Permatasari et al. examined the effect of eugenol on cell migration. Specifically, eugenol exposure to HeLa cells was given at varying doses in scratched wells [29]. In HeLa cells, eugenol boosts apoptosis. It had a cytotoxic influence on HeLa cells at concentrations of 50–200 μM. In particular, caspase-3 and p53 protein expression are involved in this apoptotic incidence [98]. One of eugenol’s anti-carcinogenic influences in cervical cancer was to hinder cell migration. As eugenol concentrations increased, cell invasion was repressed; HeLa cells exposed to eugenol (200 μM) displayed the most efficient cell migration suppression [29].

E-cadherin is a cell adhesion molecule that also serves as a marker for EMT. EMT is linked to E-cadherin expression loss, which commonly happens during cancer spread [99]. Studies were also achieved to examine the activity of methyl eugenol and cisplatin versus cervical cancer cells. The medicines were utilized discretely and in permutation. Methyl- eugenol united with cisplatin boosted the anticancer influence by triggering apoptosis and damaging HeLa cells in contrast to the medicine influences. Cell numbers in the G0/G1 phase, caspase-3 action, and mitochondrial membrane potential damage were considerably boosted in joint action contrary to the separate administration [27].

### 6.5. Melanoma

Melanoma (malignant melanoma) progresses from melanocytes and is a kind of skin cancer [100]. Amongst all types of skin cancers, melanoma is responsible for just 4%. Nevertheless, it is accountable for an elevated death rate, with over 80% of the deaths due to skin cancer [101]. Pisano et al. examined the anti-invasive influence of eugenol against melanocytes [80]. Eugenol seizes the cell cycle and induces apoptosis. In addition, Miyazawa and Hisama investigated the influence of eugenol on a B16 xenograft model [81]. Eugenol works through the synthesis of ROS [82], which leads to DNA synthesis inhibition, hence postponing cancer progress. A 40% decrease was documented in tumor size via eugenol activity [83,84]. In another study regarding the anticancer action of eugenol on human melanoma cells (WM1205Lu), eugenol showed an induction of apoptosis and arrested the cell cycle at the S-phase [83]. Ghosh et al. [85] explored eugenol and isoeugenol (an isomer of eugenol) for anti-melanoma action. They concluded that eugenol, but not isoeugenol, exhibited anticancer action against melanocytes [82].

Eugenol and six of its derivatives were examined by Pisano et al. [80] for their anti-proliferative action on primary melanoma cell lines. They deduced that the biphenyl eugenol derivative enantiomer (S)-6,6′-dibromo-dehydrodieugenol (S7-S) possesses the capability to trigger apoptosis in neuroblastoma and melanoma as compared with other eugenol derivatives. Eugenol treatment caused apoptosis of G361 cells with the probable caspases 3 and 6 association. Furthermore, substrates of caspases, such as PARP, DFF45, and lamin A, were slashed throughout eugenol-triggered apoptosis in G361 cells. Gosh et al. [85] implied that eugenol might be established as an E2F-targeting factor to treat melanoma. Eugenol hindered the proliferation of osteosarcoma (HOS) cells in both dose- and time-dependent ways. Elevated caspase-3, p53, and PARP cleavage levels are associated with eugenol-triggered apoptosis in HOS cells [86].

Nanotechnology approach has an important capacity in melanoma treatment, since it allows researchers to precisely target the tumour cells with anticancer medicines [26,102,103]. Mishra and colleagues used a solvent injection approach to create hyaluronic acid (HA)-coated liposomes that were laden with an efficient blend of dacarbazine and eugenol (antimelanoma drugs) [104]. With just a 0.5 µg/mL dacarbazine dose, coated-dacarbazine eugenol liposomes displayed 95.08% cytotoxicity as compared to 10.20% cytotoxicity by dacarbazine solution at the identical dose. Besides, coated-dacarbazine eugenol liposomes demonstrated a remarkable increased decline in cell invasion and proliferation. The suppression of the antiapoptotic protein survivin, which is highly expressed in melanoma cells and causes them resistance to apoptosis, is thought to be responsible for this performance. Eugenol addition led to survivin protein downregulation, thus allowing dacarbazine to achieve its role with dramatically increased cytotoxicity, elevated apoptosis, and significantly reduced cancer cell migration and proliferation. Therefore, surface-functionalized dacarbazine- and eugenol-laden liposomes possess substantial potential against resilient and violent metastatic melanoma [26].

Dwivedi et al. compared the anticancer actions of three distinct clove (*S. aromaticum*) extracts against varied cancer cell lines in vitro. Water, ethanol, and oil extracts were tested against various cancer cell lines, such as MCF-7 (ER+ve), MDA-MB-231 (ER−ve) breast cancer, HeLa (cervical cancer), DU-145 prostate cancer, TE-13 esophageal cancer cell lines, as well as normal human peripheral blood lymphocytes in order to evaluate their anti-proliferative activities. The MTT assay was used to assess cell proliferation inhibition. The extracts inhibited cell growth in a variety of ways in the five cancer cell lines studied, with the oil extract having the most cytotoxic effect. Cell disruption with membrane rupture caused cytotoxicity, according to morphological examination and DAPI staining. Clove oil at 300 μL/mL caused maximum cell death in TE-13 cells after 24 h, with 80 percent cell death, but DU-145 cells showed low cell death. However, no appreciable cytotoxicity was detected in human peripheral blood mononuclear cells (PBMCs) at an identical dose [105].

### 6.6. Breast Cancer

Kumar et al. studied if cloves have any cytotoxic action on MCF-7 human breast cancer cell lines. A brine shrimp lethality test (BSLT) and an MTT assay were used to examine the anticancer capability of varied doses of water extract, ethanol extract, and clove essential oil in vitro. The essential oil of cloves demonstrated the most cytotoxic influence, followed by ethanol and water extract. The LD_50_ concentration of clove essential oil in the 24 h BSLT was 37 μg/mL. Besides, the clove essential oil’s IC_50_ values in the 24- and 48-h MTT assays were 36.43 and 17.6 μg/mL, respectively. Therefore, they concluded that cloves are a prospective source for the creation of anticancer drugs [106].

Breast cancer is a serious life-threatening health problem for millions of women around the world every year. It is classified as the second most common cancer in women, and is ranked fourth for cancer-related deaths globally [37]. In women, mammary epithelial cells are controlled by keeping a balance between the cell cycle and apoptosis. Disruption of this causes an increase in mammary epithelial cells, lastly leading to breast cancer [107]. The majority of chemotherapy agents that are presently utilized as a means of treatment of this malady are extremely lethal and have long-term side effects. Thus, new anticancer medicines with greater efficacy and specificity are immediately required. The influence of eugenol on apoptotic and procarcinogenic proteins, both in vitro and in tumor xenografts, was evaluated via immunoblotting and RT-PCR was utilized to determine the influence of eugenol on E2F1 and survivin mRNA levels. Al-Sharif et al. examined the influence of eugenol on cell proliferation by means of a real-time electronic cell detection system. Low-dose eugenol (2 µM) has explicit toxicity against several breast cancer cells. This lethal influence was chiefly facilitated by induction of the internal apoptotic pathway and potent downregulation of E2F1 and its downstream antiapoptosis target, surviving, regardless of p53 and ERa status. Eugenol also inhibited many other breast cancer-related oncogenes, like NF-κB and cyclin D1. Furthermore, eugenol upregulated the multipurpose cyclin-dependent kinase inhibitor p21^WAF1^ protein and inhibited the proliferation of breast cancer cells in a p53-independent manner. Significantly, these antiproliferative and pro-apoptotic influences have also been noticed in vivo in xenografted human breast cancers. Therefore, eugenol displays anticancer characteristics both in vitro and in vivo, demonstrating that it might be utilized to strengthen adjuvant breast cancer treatment by targeting the E2F1/surviving pathway, particularly for the less-sensitive triple-negative subtype of the disorder [87]. Abdullah et al. investigated the probability of using eugenol as an antimetastatic and antiproliferative compound against MDA-MB-231 and SK-BR-3 breast cancer cells. Treatment with 4 and 8 μM eugenol for 48 h substantially hindered cell proliferation of MDA-MB-231, with an inhibition rate of 76.4%, while 5 and 10 μM of eugenol for 48 h substantially hindered the proliferation of SK-BR-3 cells with an inhibition rate of 68.1%. Eugenol-treated cells demonstrated substantially reduced expression of MMP2 and MMP9 and an insignificant rise in the expression of TIMP1 in HER2-positive and triple-negative breast cancer cells. Eugenol greatly raised the proportion of MDA-MB-231 and SK-BR-3 cells in late apoptosis and elevated Caspase3, Caspase7, and Caspase9 expression [88]. Vidhya and Devaraj [108] verified that breast cancer cells (MCF-7) experience the potent antimutagenic action of eugenol. Eugenol is both time and dose dependent when defeating the proliferation of MCF-7 cells [108,109]. Furthermore, Pisano et al. [80] described the antiproliferative activity of eugenol-related biphenyl (S)-6,60-dibromo-dehydrodieugenol, via induction of apoptosis.

**Figure 3:**
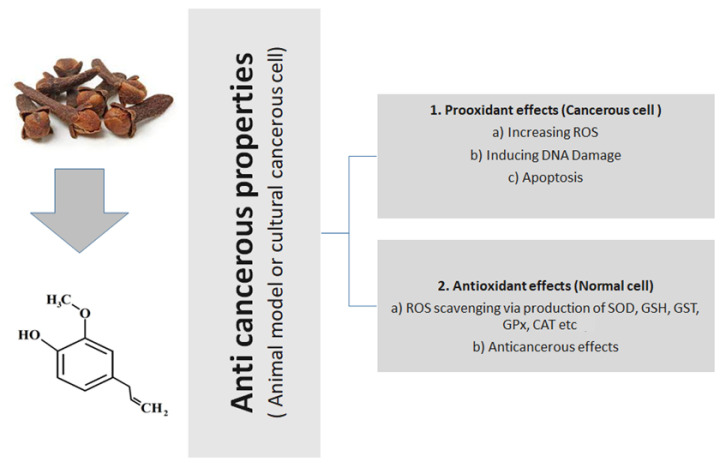
General anticancerous action of eugenol (tried and tested in animal or cultural cancerous cell models).

The modification of autophagy may enhance either the survival or apoptosis of cancer cells. Abdullah et al. treated triple-negative (MDA-MB-231) and HER2-positive (SK-BR-3) breast cancer cell lines with varied eugenol doses. They examined apoptosis via a flow-cytometry technique, whereas autophagy via acridine orange. They investigated the influence of eugenol on the gene and protein expression levels of autophagy and apoptotic genes. Treating cells with varying doses of eugenol substantially hindered cell proliferation. The protein levels of AKT serine/threonine kinase 1 (AKT), forkhead box O3 (FOXO3a), cyclin-dependent kinase inhibitor 1A (p21), cyclin-dependent kinase inhibitor (p27), and Caspase-3 and -9 increased considerably in eugenol-treated cells. Furthermore, eugenol triggered autophagy through upregulation of microtubule-associated protein 1 light chain 3 (LC3) expression levels and downregulation of nucleoporin 62 (NU p62) expression. Eugenol is a potential natural anticancer agent against triple-negative and HER2-positive breast cancer. It seems to act via targeting of the caspase pathway and via triggering of autophagic cell death [28]. Figure 3 presents the general action of eugenol, tried and tested in animal or cultural cancerous cell models.

## 7. Combination Therapies

Drug combination remedies are generally utilized in fighting cancers. Eugenol demonstrates a synergistic influence when utilized with certain chemoinhibitory drugs, resulting in a significant decrease in drug injury to normal cells. The use of eugenol synergistically strengthens the influence of gemcitabine [30]. Several studies have also assessed the effectiveness of eugenol alone or in combination with other medicines according to these findings. Hussain et al. [32] exhibited that eugenol alone inhibited cell proliferation and boosted treatment efficiency when coupled with gemcitabine against HeLa cells, a human cervical tumor cell line. The side effects caused by gemcitabine treatment can be reduced by eugenol. These advantageous influences seem to be mediated through its antiapoptotic and anti-inflammatory influences, because they were linked with increased caspase-3 action and decreased COX-2 and IL-1β expression, respectively [32]. Moreover, another study stated eugenol boosts cytotoxicity against triple-negative breast cancer cells (TNBCs) and animal models, as well as having synergistic chemotherapeutical influences with cisplatin. The suppression of the NF-B signaling pathway, which led to phosphorylation of the p50 and p65 subunits and, as a result, invasion of the cellular nucleus, decreased the levels of IL-6 and IL-8, which was a crucial factor in this influence. Thus, eugenol boosted the cytotoxic and pro-apoptotic actions of cisplatin [33]. Eugenol is thought to help cisplatin suppress breast cancer stem cells by hindering the action of aldehyde dehydrogenases (ALDH) and ALDH-positive cancer beginning cells, as well as inhibiting the NF-B signaling pathway. These findings imply that a treatment combining eugenol and cisplatin could be an efficient cure for TNBC. In the same way, the susceptibility of a human immortal cell line from HeLa (cervical cancer) to cisplatin seems to be upsurged by eugenol [55]. A combination of cisplatin and eugenol was more effective in cell growth inhibition than using cisplatin alone. Together, these findings imply that the use of medications in combination can boost their therapeutic efficiency against cancer cells. Eugenol was efficient as an anticancerous agent under lab conditions, given alone, or in connotation with orthodox remedies like doxorubicin [76,77].

## 8. Eugenol Toxicity

The toxicity of eugenol has been examined in many in vivo studies. Nevertheless, slight information is accessible in humans [110]. Eugenol toxicity is principally concentration dependent [22]. Eugenol’s prooxidant influence causes its toxicity [111]. Medeiros et al. [112] stated that eugenol’s toxicity is ascribed to protein deactivation because of eugenol binding at the lysine residues. The cytotoxicity of eugenol is probably a result of its metabolic responses. Then, the responsive metabolites react more with DNA, making complexes that may damage nuclear genomic material. Eugenol is distinguished as a contact allergen in dental medicine [110] and causes its entrance into the circulation via permeating dental pulp tissue, leading to chromosomal abnormalities in the cells of dental pulp [113].

Eugenol is recognized for its anti-inflammatory, antioxidant, anticancer, and antibacterial properties; nevertheless, relying on the histologic structure exposed to eugenol and the dose utilized, it may cause toxicity [114]. At doses ranging from 0.06–5.1 μM, the toxicity of eugenol was noticed in human dentistry and the oral cavity at increased interval doses of 320–818 μM [115]. In addition, at all doses examined (0.62, 1.24, and 2.48 mg/mL), eugenol was capable of activating genotoxicity via triggering DNA damage in mouse peritoneal macrophages. Though, it has exhibited antigenotoxic capability relying on the handling procedure, which might be interconnected with its influence on drug metabolism [85]. Thus, eugenol may modify oxidizing and inflammatory processes. Nevertheless, its utilization should be made consistent with the remedial safety demonstrated in toxicity. A few studies in humans have reported the toxic effects of accidental exposure to eugenol in the liver, lung, and nervous system as discussed earlier in this review. Overall, the toxic effect of eugenol on mammals is low, and the US Environmental Protection Agency has categorized eugenol as category 3. The oral LD_50_ value is >1930 mg kg^−1^ in rodents as given in Table 2 [116].

## 9. Conclusions

Phenolic phytochemicals are a wide group of nutraceutical ingredients found in plants. They have been broadly studied owing to their health-enhancing capacity. For example, eugenol (4-allyl-2-methoxyphenol; C_10_H_12_O_2_) is a very vigorous constituent of clove. In addition, musky plants, such as cinnamon, basil, bay leaves, and nutmeg, comprise eugenol. Eugenol has many applications, such as in medicine, perfumery, flavorings, and essential oils. A growing body of information has shown that eugenol has anti-inflammatory, antioxidant, anticancer, antimutagenic, and antigenotoxic characteristics. The anticancer activities of eugenol in several cancer types have been well recognized. The present review highlights the antiproliferative action and molecular mechanisms of eugenol-induced apoptosis against different cancer cells. Although large amounts of eugenol can be pro-oxidative and hazardous, the FAO considers concentrations beneath 2.5 mg/kg body weight to be secure. In some circumstances, particularly among dental practitioners, eugenol can induce allergies, for example, allergic contact dermatitis. Eugenol has numerous uses because of its diverse biological properties. Several studies showed evidence of eugenol’s capability to treat several ailments. As a consequence of the presence of eugenol in certain medicinal plants, the usage of these plants may lead to health advantages and thus an enhancement in life quality. Eugenol has exhibited remedial capability in medicines, involving those planned to protect against varied cancers. Furthermore, several drugs have a synergistic influence with eugenol. Low response rates restrict conventional chemotherapy and immunotherapy and display no improvement in overall survival. Amongst many adopted approaches, nanoparticles that grasp the substantial potential for improving the delivery of antitumor drugs are promising since they can raise the effectiveness of eugenol while simultaneously decreasing the adverse influences of conventional formulations.

## Figures and Tables

**Table 1:** Anticancerous potential of eugenol against several cancer types.

Type of Tumor	Type of Study	Effective Dose	Mode of Action	References
Lung cancer	in vitro	low concentrations to 1000 μM	reduces cyclooxygenase-2 activity, promotes cell cycle arrest at S-phase and initiates apoptosis	[20,55]
Colon cancer	in vitro	800 µM	cell death, necrosis, and slows down cell cycle. Eugenol synergistically boosts the cytotoxic and pro-apoptotic actions of cisplatin, doxorubicin and cinnamaldehyde	[25,76,77]
Gastric cancer	in vitro	low concentration of eugenol loaded with chitosan nanopolymer	stops cancer development, up-regulation of proinvasive and angiogenic factors, favors apoptosis by the mitochondrial pathway through modulating Bcl-2 family proteins	[78,79]
Cervical cancer	in vitro	50–200 μM	boosts apoptosis, cell migration suppression at high concentration	[29,40]
Melanoma	in vitro	0.5 µg	Stops cell cycle and triggers apoptosis, inhibits DNA synthesis	[80,81,82,83,84,85,86]
Breast cancer	in vitro and in vivo	2 µM	down regulating E2F1 and its downstream antiapoptosis target; inhibits breast cancer-related oncogenes	[87,88]

**Table 2:** LD_50_ values of eugenol in laboratory animals.

Species	Dose LD_50_/LC_50_
Rat	LD_50_ oral: 1190–2680 mg kg^−1^
Mice	LD_50_ oral: 3000 mg kg^−1^
Mice	LD_50_ intraperitoneal: 500–630 mg kg^−1^
Guinea pig	LD_50_ oral: 2130 mg kg^−1^

## Data Availability

Data are contained within the article.
